# Factors Influencing Bank Geomorphology and Erosion of the Haw River, a High Order River in North Carolina, since European Settlement

**DOI:** 10.1371/journal.pone.0110170

**Published:** 2014-10-10

**Authors:** Janet Macfall, Paul Robinette, David Welch

**Affiliations:** Center for Environmental Studies, Elon University, Elon, North Carolina, United States of America; Chinese Academy of Sciences, China

## Abstract

The Haw River, a high order river in the southeastern United States, is characterized by severe bank erosion and geomorphic change from historical conditions of clear waters and connected floodplains. In 2014 it was named one of the 10 most threatened rivers in the United States by American Rivers. Like many developed areas, the region has a history of disturbance including extensive upland soil loss from agriculture, dams, and upstream urbanization. The primary objective of this study was to identify the mechanisms controlling channel form and erosion of the Haw River. Field measurements including bank height, bankfull height, bank angle, root depth and density, riparian land cover and slope, surface protection, river width, and bank retreat were collected at 87 sites along 43.5 km of river. A Bank Erosion Hazard Index (BEHI) was calculated for each study site. Mean bank height was 11.8 m, mean width was 84.3 m, and bank retreat for 2005/2007-2011/2013 was 2.3 m. The greatest bank heights, BEHI values, and bank retreat were adjacent to riparian areas with low slope (<2). This is in contrast to previous studies which identify high slope as a risk factor for erosion. Most of the soils in low slope riparian areas were alluvial, suggesting sediment deposition from upland row crop agriculture and/or flooding. Bank retreat was not correlated to bank heights or BEHI values. Historical dams (1.2–3 m height) were not a significant factor. Erosion of the Haw River in the study section of the river (25% of the river length) contributed 205,320 m^3^ of sediment and 3759 kg of P annually. Concentration of suspended solids in the river increased with discharge. In conclusion, the Haw River is an unstable system, with river bank erosion and geomodification potential influenced by riparian slope and varied flows.

## Introduction

Streams and rivers are considered to be in a state of dynamic equilibrium when the sediment delivered to the channel is in balance with the capacity of the stream to transport and discharge that sediment [1]. Stream channels alternatively experience periods of alluvial deposition, followed by erosional downcutting of the alluvium, followed by periods of additional deposition. These cycles have created a landscape of terraces and floodplains, sculpted by the streams and rivers flowing through them [2]. Globally, changes in land use, climate and other factors have altered the historic patterns of transport and discharge, with significant changes to river shape, processes, sediment dynamics and water quality [2]. With these changes, soil erosion has been identified as a significant challenge in both developing and developed countries.

A landscape perspective of rivers and their watersheds demonstrates the influence of land use and disturbance on river structure and ecology at multiple scales [5]. European settlement in the southeastern United States began a period of forest clearing in the 1700's, followed by row crop agriculture [3]. These practices had deleterious ecological consequences to surface waters in the form of increased sediment loads and habitat degradation [3], [4], [6]–[9].

Before urbanization and agricultural clearing, streams in the Piedmont region of the southeastern United States, had low levels of suspended solids and high connectivity between the stream and surrounding floodplains [3], [4]. It has been estimated that 25 km^3^ of soil have eroded from agricultural lands in the Piedmont region between the coastal plain and the Appalachian Mountains, with an average of 14 cm of topsoil lost from North Carolina since the early 1700's [3]. This erosion from agriculture has left a legacy of upland gullies and sediment deposition near and in streams and rivers [3].

Another anthropogenic disturbance to streams and rivers is the proliferation of dams and artificial water bodies. There are over 2 million artificial surface water impoundments including 82,000 dams in the continental United States, with some dating from the 17^th^ century [10], [11]. Both natural and man-made dams can have profound impacts on the ecology and geomorphology of rivers, altering patterns of sediment transport and deposition, water and energy flow, and aquatic habitat [10], [12], [13]. Downstream channel degradation due to dams has been documented for more than 85 years and in many cases has been extreme [14]–[17]. Downstream changes often include channel incision, channel pattern change (braided to single-thread or vice-versa), loss or encroachment of vegetation, and bank collapse [13], [18], [19]. Upstream of the dam there may be sediment deposition within the impoundment, leading to incision when the dam is removed [20].

Upstream urbanization, with the increase in impervious surface, has been shown to alter the flow of streams, increasing the frequency of “flashiness” and bank incision [2]. Susceptibility to erosion and hydromodification from urbanization varies with stream bed composition, armoring, bank height, bed and bank materials, precipitation patterns, and other factors [21], [22].

Sediment loading from bank erosion is a land management problem of global importance [23], [24]. Sediment is one of the most common pollutants from non-point sources, with over 6,000 water bodies across the United States showing significant suspended sediments [25]–[27]. Different bank materials, aerial and subaerial weathering, variations in grain size, shear strength of the bank materials, bank angle, and water potential can influence river bank mass wasting, failure and fluvial entrainment [23]–[25]. The ability to predict bank failure and erosion, however, is often uncertain, especially for stream banks with a varied depositional history such as from anthropogenic disturbance [26].

Processes that determine channel geomorphology differ between first and second order streams and larger rivers with larger watersheds [27]–[29]. Few high order rivers have been studied [27], [30]. In large rivers, conditions leading to river widening often are nonlinear, with energy adjustment resulting in different and sometimes opposite adjustment processes. This was seen in the North Fork Toutle River system in Washington State in the NW United States, where following the eruption of Mount St. Helens, one river was dominated by aggradation and widening, while another similar river was dominated by degradation [31]. Unstable channels continue to adjust following major disturbances, both anthropogenic and natural, until a stable floodplain is established with a progressive armoring of the channel bed [32], [33].

Sediment and nutrient loading are a global water quality concern, and are significant issues in the Haw River, a high order river in the North Carolina Piedmont. Jordan Reservoir, a major drinking water supply, is formed by a dam on the Haw [34]. However, water in the reservoir is considered impaired due to algal blooms from excess nutrients. To improve water quality, nutrient reduction goals have been established. Understanding the patterns of geomorphic change of the river and contributions of the river bank to sediment and nutrient load may provide a model for water quality improvement in this and similar systems.

Similarly to other developing areas, the Haw River watershed has a history of profound disturbance through forest conversion to row crop agriculture, the construction of dams, and upstream urbanization. In 2014, it was identified as the 9^th^ most threatened river in the United States by the organization American Rivers. The river today has little resemblance to the clear water and low banks from historical descriptions [35].

The primary objective of this study was to identify factors influencing bank geomorphic change and erosion of the Haw River using field measurements. Few reports on erosion of high order rivers have been published, with most based on model estimations. River traits included river slope, riparian soil type and slope, land cover, bank angle, surface protection, bank height, bankfull height and root density and depth. The Haw River has highly incised, unstable banks exhibiting extensive mass wasting, undercutting with bank collapse and fluvial entrainment. Understanding alluvial channel behavior and the channel response to disturbance will provide insight into understanding the factors controlling erosion patterns, shape and balance of the Haw and other high order rivers.

## Materials and Methods

For study sites that were located on public lands, the field sites were located in local parks (town parks). Local government agencies were project partners and did not require permits for access. For sites that were located on private land, we obtained landowner permission for access. However, most landowners in our region are reluctant to allow access to their lands by strangers and requests for access may result in alienation of landowners. This research is being used as the foundation for development of the Haw River Trail - a project which uses recreation to achieve regional conservation goals. Because of the trust and contacts established with the research project described in the submitted paper, landowners have been willing to work with us and with local governments on this conservation/recreation project. Alienation of landowners would compromise progress on the conservation work we are developing on the Haw. When we made contact for this study, some of the landowners requested that their identities not be made public. Landowners highly value the personal connections that were established with this project, which is contributing to the success of the conservation work. However, landowners in this region also highly value their privacy and private property rights.

We will share an excel spreadsheet with the GPS coordinates through requests to the senior author. The authors will assist landowner contacts if requested.

**Figure 1:**
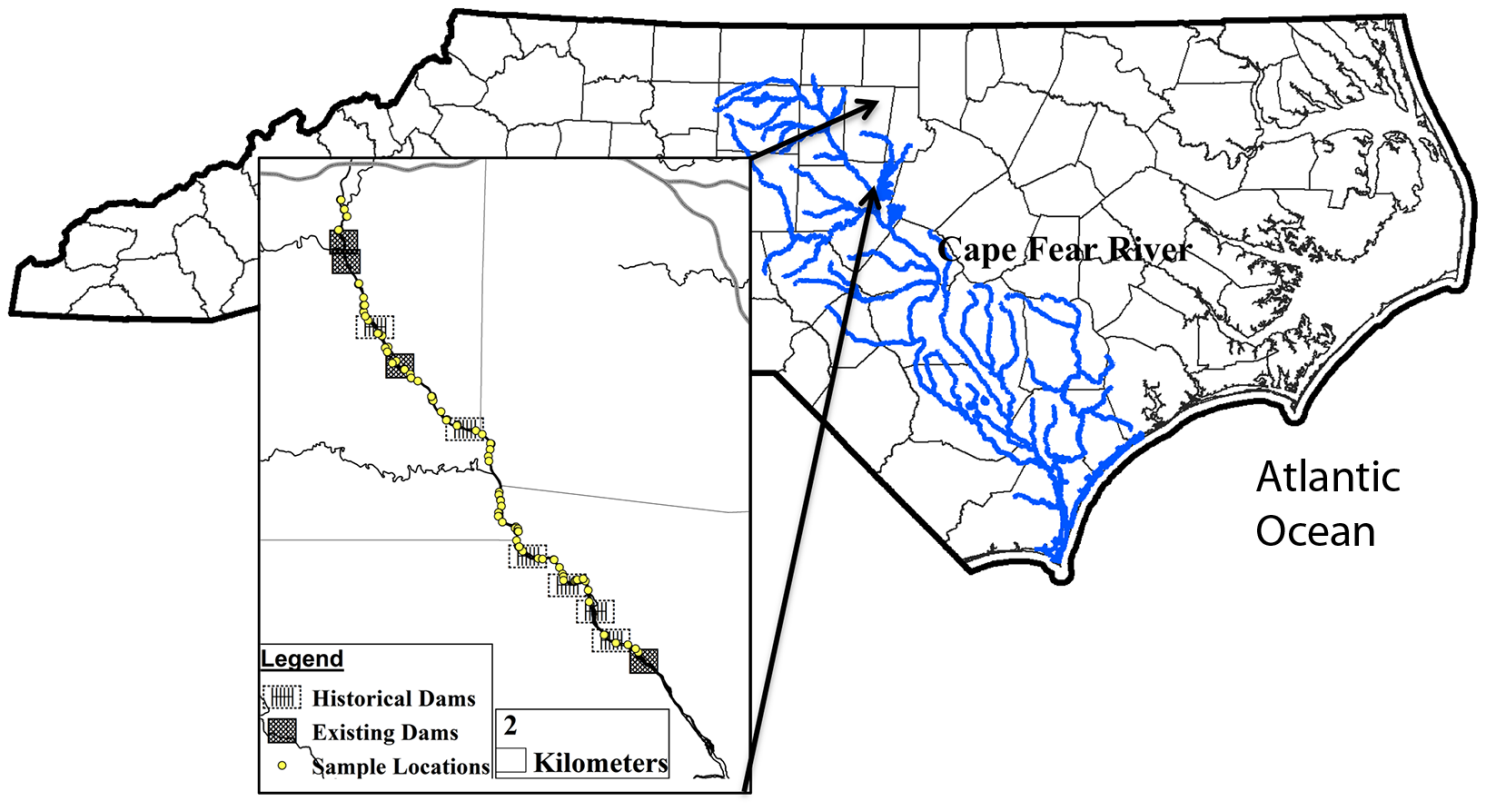
Location of the study area on the Haw River.

The Haw River is located in the north central Piedmont region of North Carolina (Figure 1). This area is located between the coastal plain to the east, and the Appalachian Mountains to the west. North Carolina borders the Atlantic Ocean in the southeastern United States. The river is approximately 177 km long with a watershed of about 3952 km^2^, including agricultural, forested, urban and suburban land cover. Nearly one million people live within the Haw River watershed, including the urbanized areas of Greensboro, Graham, Elon, and Burlington, North Carolina which are all upstream of the study reach.

The Haw River ends at the confluence with the Deep River near Moncure, North Carolina, forming the headwaters of the Cape Fear River. For most of its length, including the section studied, the river is relatively straight with few meanders. The watershed is characterized by low, gently rolling hills. Low naturally formed levees (typically ≤1.5 m in height) are frequently found on the river bank. Behind the river bank is a backswamp of varied width. Behind the backswamp is the toe slope, extending to the upland areas. The river channel bottom ranges from bedrock to mixed sand/silt/cobbles with large woody debris. The river is a riffle – pool system [36]. Mean discharge at the United States Geological Survey monitoring station near Bynum, North Carolina (1973–2012) immediately downstream of the study area was 34 m^3^/s, with discharge ranging from 1642 m^3^/s (1996) to 0.005 m^3^/s (1983) [37]. Elevation ranged from 97m at the lower end of the study area to about 304 m at the top of the watershed upriver of the study section.

The study area extended 43.5 km, from the intersection of the Haw River and Interstate 40/85 in Burlington, North Carolina to the intersection of the river and US 15-501 in Bynum, North Carolina, flowing through the counties of Alamance, Orange, and Chatham. The upriver location beginning the study area was approximately 112 km downstream from the headwaters and 65 km upstream from the confluence of the Haw River and the Deep River. The studied portion of the river is a fourth order river and higher [38]. Data were collected from 87 bank sites within the study area. Sites were accessed by kayak and land. (Figure 1).

An opportunistic sampling strategy for study site selection which was contingent upon landowner permission was used. Most study sites were on privately owned land. Study sites that were on public land were located in local parks where the local governments were project partners and no permits were required. Eighty-seven river sections that had more than 6 m in length of relatively homogenous slope, bank angle, bank height, and vegetation cover were selected for study. There were no endangered or protected species present at any of the study sites. Measurements were collected during 2006–2007, primarily during spring, summer and fall.

The geographic locations of the bank sites were documented using a Magellan handheld GPS. The lengths of the banks with homogenous characteristics were measured using a Leica Laser Distometer and heights were determined using a telescoping measuring rod and the distometer. The sites were also photographed and sketched to record the appearance of toppled trees and bare soil.

### Bank Erosion Hazard Index Methods

A Bank Erosion Hazard Index (BEHI) was used to assess the potential stability of the Haw River's banks [39], [40]. This method estimates and predicts erosion potential of rivers and streams, providing documentation of geomorphic field parameters and channel adjustment predictions. While the BEHI methodology is often a small component of more extensive river geomorphic assessments, it is a convenient and useful tool to rapidly create an inventory of river bank characteristics and to assess river bank and river adjustments. Rosgen's BEHI method is normally used to assess lower order rivers and is not often used on large rivers, such as the Haw River [39].

The BEHI is based on five measurements related to bank erosion potential (Table 1). Measurements were conducted according to methods outlined in Rosgen [39], [40]. The bank height was measured at the top of the vertical or sloped area rising from the channel bed. Rooting depth was measured across the bank surface. Root density was determined from a visual estimation of the surface coverage across the bank surface. Surface protection was determined from the percentage of the bank with root, vegetation or hardened structure coverage. From these field measures, bank height ratio (bank height/bankfull height) and root depth ratio (root depth/bank height) were calculated [39].

**Table 1:** River bank erosion metric ranking scores for calculation of the BEHI.

Category		Bank Ht. Ratio (m/m)	Root Depth Ratio (%)		Root Density (%)	Bank Angle (Degrees)	Surface Protection (%)	Total Index	
Very Low	Value	1.0–1.1	100–80		100–80	0–20	100–90		
	Index	1–2	1–2	1–2		1–2	1–2		≤10
Low	Value	1.1–1.2	80–55	80–55		20–60	90–50		
	Index	2–4	2–4	2–4		2–4	2–4		10–20
Moderate	Value	1.2–1.5	55–30	55–30		60–80	50–30		
	Index	4–6	4–6	4–6		4–6	4–6		20–30
High	Value	1.5–2.0	30–15	30–15		80–90	30–15		
	Index	6–8	6–8	6–8		6–8	6–8		30–40
Very High	Value	2.0–2.8	15–5	15–5		90–120	15–5		
	Index	8–9	8–9	8–9		8–9	8–9		40–45
Extreme	Value	>2.8	<5	<5		>120	<5		
	Index	10	10	10		10	10		>45

Table 1 lists criteria used for calculation of the BEHI measurements (Table 1). In the Very Low erosion risk category, a value of between 1–2 is assigned. For example in a low risk stream, the Bank Height Ratio is low, there is extensive rooting in the bank (80–100% of the bank), there is high root density, low bank angle, and substantial surface protection, Summing the BEHI index values for the physical and biological traits gives a total of 5–10, indicating a low potential for erosion. In contrast, attributes that would suggest extreme risk of erosion (the bottom row) include high banks, few roots extending down the bank with low root density, an undercut riverbank with high angle, and little surface protection.

A BEHI value was calculated at each studied site on the river based on Rosgen's BEHI method [40]. A value of 1 (very low) to 10 (extreme) was assigned to each of the bank metrics, as indicated in Table 1. Numbers for all traits were summed for each site. A total value of <10 was considered a very low bank erosion hazard index. A value> 45 was considered an extreme bank erosion hazard index.

Identification of the bankfull stage can be difficult to determine for rivers with an unstable channel. Rather than presenting a stable state, rivers are open systems which respond to variations in energy and materials, making bankfull assessment sometimes uncertain [31]. In cases where the bank height exceeded the bankfull height, the bankfull height was measured at the place on the river bank with visible vegetation changes indicating plants experienced flooding, such as a change in root morphology or root washout [26], [27], [41]. Where roots were not present, the bankfull height was measured from the point on the bank where bank materials showed evidence of saturation or shear force stress from flowing water.

Soils were sampled for nutrient analysis by bulk density sampler from the top 20 cm of the river bank at 31 locations throughout the study area. Duff was removed and soils were air dried and analyzed for bulk density and nutrient content by the NC Department of Agriculture and Consumer Services.

### River Width and Slope Calculation Methods

The river width was measured using a geographic information system (ArcGIS, ESRI, Inc.) to analyze aerial photographs of the study area. Aerial photographs for Alamance, Orange (2005) and Chatham counties (2007) were obtained from the county GIS offices. Aerial photographs for Alamance and Orange (2011) were from DigitalGlobe (supplied through ESRI, Inc., Redlands, CA) and for Chatham Co. (2013) through NCOneMap. Widths were determined from the aerial photographs by measuring a line placed perpendicular to the center line of the river. The river width measurements were taken of the visible water surface, representing the river width at base flow. The river was at base flow at all dates of image acquisition. Digital resolution was 0.27 m. Locations of dams which are still present in the river were recorded from these aerial photographs. To confirm consistency of measurements, lengths of 10 hardened structures (dams, bridges, buildings) were measured in 2005/2007 and 2011/2013 images. There were no differences in measurements between the two image sets for hardened structures.

The slope of the river was estimated from Digital Elevation Models obtained from the NC Floodplain Mapping Program, 2013. Digital vertical resolution was 0.25 m and horizontal resolution was 6 m. A center line was established on the river image and points were placed every 500 meters. The river slope was measured for the entire length of the study section of the river.

### Historical Dam Locations

The locations of historical dams, which are no longer present on the river, were determined by comparing the GIS data layers with photographs and topographic maps. Locations of historical dams were estimated from U.S. Geological Survey topographic quadrangles (scale 1∶24,000) and historical records [42]–[44](Figure 1).

### Soil and Land Cover Methods

Aerial photographs from 2005/2007 were used to digitize different types of land cover within 153 meters (500 ft.) of both of the river banks for evaluation of the riparian corridor over the entire river length studied. Within the GIS, polygons were drawn around each type of designated land cover. The areas for these polygons were then calculated and summed for each land cover category. The land cover classes were:

Forest – areas with evident canopy coverage of mature trees

Open - areas lacking trees or shrubs

Shrubland – areas dominated with small-canopied vegetation

Impervious – areas such as roads and buildings

Water – streams, ponds and other water features

Soil type and traits at each study site were determined from the Alamance, Orange and Chatham Co. Soil Survey. Soil maps (SSURGO) were obtained from the Natural Resource Conservation Service Web Soil Survey [45]. Locations for each study site were identified in the Soil Surveys using GIS. Soil types were determined for soils immediately adjacent to each study location, using the GIS shapefiles for soils in each county. Slopes which are characteristic for each soil type adjacent to the study sites were recorded based on the SSURGO soil type descriptions.

### Statistical Analysis

Statistical analyses were conducted with SAS Enterprise Guide 4.3 (SAS Institute, Cary, NC). A Pearson product moment correlation analysis was calculated to determine independence of the river attributes and erosion potential. Analysis of variance or a Whitney Mann Rank Sum Test was calculated to determine effect of riparian slope and historical dams. Data used in these analyses is freely available at http://www.elon.edu/e-web/academics/elon_college/environmental_studies/macfall-plos-one.xhtml.

## Results

### Bank Erosion Hazard Index

A majority, 84% percent, of the studied banks had a BEHI value of moderate to high erosion potential (Table 2). The mean bank height was 11.8±0.5 m and the mean bankfull height was 5±0.1 m. The mean bank angle was 53°, with a maximum of 90°. The river had widened (bank retreat) by 2.3 m over the six year period from 2005/2007 to 2011/2013, suggesting rapid change to the river bank and ongoing erosion (Table 2).

**Table 2:** Summary statistics for bank characteristics of the Haw River.

Attribute	Mean	SE	Min	Max	Median
River Width 2005/2007 (m)	84.3	5.7	27.4	300.5	64.8
River Width 2011/2013 (m)	86.7	5.6	27.4	300.2	68.6
Bank retreat (m)	2.3	0.3	−1.4	9.5	1.5
Riparian slope (%)	6.6	0.9	1	30	1
Bank angle	53.2	2.5	3	90	55
Elevation (m)	122.7	1.4	97	145.7	120.4
River bed slope (%)	0.08	0.01	0	0.37	0
Bank height (m)	11.8	0.5	1.8	29.8	12.1
Bankfull height (m)	5.0	0.1	1.8	9.2	5
Bank height ratio	2.4	0.09	1	4.2	2.4
Root depth ratio	0.59	0.04	0	1.0	0.6
Surface protection (%)	35	2.4	4.3	83.4	30
Root density (%)	43	2	0	100	45
BEHI	24.3	0.7	9.1	39.6	24.1
**BEHI Category**	**Very low**	**Low**	**Moderate**	**High**	**Very High**
Number of Sites	1	13	58	15	0
Percent of Sites	1.2	14.9	66.7	17.2	0
Total sites	87				

### River Width and Slope

**Figure 2:**
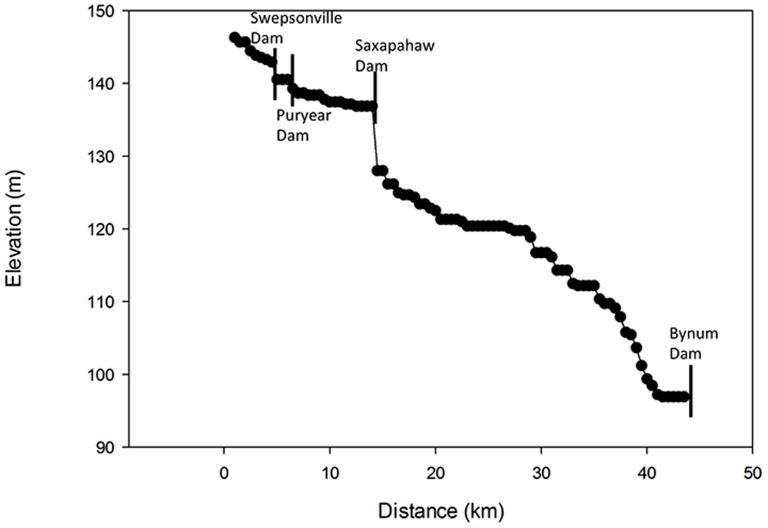
Longitudinal profile of the Haw River study site.

The slope of the river varied throughout the study area, with some sections showing little drop and others a much steeper gradient over a short run (Figure 2). Upstream of the Saxapahaw Dam (9.1 m in height), the highest dam in the river segment studied, the river was 300 m wide, its widest point in the study segment (Table 2).

A number of significant correlations between the river bank geomorphology and erosion potential with attributes of the studied sites were noted (Table 3). There was a significant negative correlation between river width with bank height and BEHI, suggesting that as the river widens, the bank height and BEHI decrease.

**Table 3:** Pearson Product-Moment Correlation Analysis of Erosion Factors on the Haw River.

Attribute		Bank height (m)	Bankfull height (m)	BEHI	Bank Retreat (m)
Bank angle	r	−0.04	−0.04		0.11
	p	0.7	0.7		0.29
Bank height (m)	r		**0.55**		−0.04
	p		**<0.001**		0.7
Bank height ratio	r	**0.76**	−0.03		−0.07
	p	**<0.001**	0.76		0.49
Bank retreat (m)	r	−0.04	0.10	0.11	
	p	0.7	0.38	0.34	
River width 2005/2007 (m)	r	**−0.68**	**−0.48**	**−0.40**	−0.09
	p	**<0.001**	**<0.001**	**<0.001**	0.37
Riparian slope (%)	r	**−0.341**	−0.15	**−0.50**	**−0.25**
	p	**<0.001**	0.16	**<0.001**	**0.02**
River bed slope (%)	r	0.02	**0.21**	−0.04	0.01
	p	0.9	**0.05**	0.73	0.9
Surface protection (%)	r	**−0.19**	−0.10		−0.002
	p	**0.08**	0.35		0.98
Root density (%)	r	**−0.36**	−0.17		0.004
	p	**<0.001**	0.12		0.96
Root depth ratio	r	**−0.29**	**−0.26**		−0.16
	p	**0.006**	**0.02**		0.14

One of the most significant results was that the slope of the riparian soils adjacent to the river bank was negatively correlated with bank height, BEHI and bank retreat. The BEHI, bank retreat and the bank heights were significantly higher at study sites with low riparian slope (<2%) compared to riparian areas with greater slopes (Table 4), suggesting greater erosion and erosion potential in areas with low riparian slope. The flatter the land adjacent to the river banks, the more the river had widened, the greater the height of the river banks, the greater the erosion potential (BEHI) and the more erosion had occurred through river widening.

**Table 4:** Analysis of Variance comparing bank characteristics between areas with low (<2%) and high (≥ 2%) riparian slopes.

Traits/Location	n	Median values (m)	Mean values (m)	SE	P*
**Bank retreat**					
Low slope	61	2.1	2.7	0.31	0.03
High slope	26	0.8	1.5	0.45	
**Bank height**					
Low slope	61	12.7	12.8	0.42	0.002
High slope	26	10.4	9.6	1.23	
**Bank angle**					
Low slope	61	60.0	56.1	2.60	0.06
High slope	26	40.0	46.1	5.35	
**BEHI**					
Low slope	61	26.5	26.3	0.70	<0.001
High slope	26	20.6	19.5	1.08	

*Mann Whitney Rank Sum Test was used for Bank retreat and Bank height analyses. ANOVA was used for BEHI and Bank Angle analyses.

Rooting patterns were negatively correlated with bank heights. There are likely two reasons for this observation. First is the protective effect from erosion which has been described from vegetation. A second reason, however, is if bank collapse or slumping had occurred, trees on the river bank would have also moved downward with the soil, increasing both the root depth ratio and root density lower in the river bank face.

In contrast to the correlation with bank height, root density and root depth ratio were not correlated with bank retreat. Areas with measureable erosion and river widening were also independent of river physical attributes including bank height, bank angle and the BEHI (Table 3).

Based on river bank height and bank retreat measurements, the amount of soil lost through erosion can be estimated for the six year period between 2005/2007 and 2011/2013. The mean value for annual soil loss from the 43.5 km section of the river which was studied was 205,320±23,000 m^3^ per year. The bulk density measurement of the riparian soil adjacent to the river was 1.01. Based on this bulk density measure, 2.3 * 10^8^ kg of soil are lost annually from the studied river segment. The P concentration from soil nutrient analyses was measured to be 17.6±1.0 mg/L. Since P generally does not leach through the soil profile and is primarily retained in the surface, if P loading to the river with erosion is only from the top 20 cm of soil; approximately 3759 kg (8286 lb.) of P enters the river annually with sediment [46].

### Historical Dams

Historically, there have been ten dams along the river reach studied, three of which still exist. The only two dams still providing hydroelectric power are the Saxapahaw Dam and the Bynum Dam (Table 5). There were low BEHI values and bank heights upriver of and closest to the Bynum and Saxapahaw Dams, as the sites were adjacent to upper reaches of the pools formed behind the dams. BEHI values and bank heights indicating severe erosion were measured downstream of the Saxapahaw dam. No measurements were made downstream of the Bynum dam, the downstream limit of this study.

**Table 5:** Historical and current dams on the Haw River listed in order on the river.

Dam Name	Dam Height (m)	Years
Virginia Falls Dam	3.0	1874- present, removed 2013
Puryear Dam	2.4	1763 - present
Cedar Cliffs Dam	1.5	1860–1910
Saxapahaw Dam	9.1	1938 - present
Dark's Dam	-	1790–1875
Elliot's Falls	1.2	1778–1810
Love's Dam	-	1790–1920
Pace's Dam	2.4	1789–1924
Burnett-Powell Dam	-	1776–1880
Bynum Dam	3.0	1874 - Present

Dams which are no longer present on the Haw River had little effect on river bank height, BEHI estimates of erosion potential or bank retreat. While it is generally accepted that sediment is deposited upstream of dams in the impoundments, the presence of historical dams on the Haw River does not appear to be a strong factor influencing current conditions (Table 6). In addition, the heights of the dams (with the exception of Saxapahaw Dam) were all lower than the mean values for bank heights measured in the current study. Impacts will likely be much greater with the remaining dams in Saxapahaw and Bynum, however, which are larger.

**Table 6:** Effect of historical dams on river bank geomorphology.

Traits/location	n	Median values (m)	Mean values (m)	SE	p
**Bank retreat**					
Behind dam	8	1.8	2.3	0.87	0.94
Not behind dam	79	1.5	2.2	0.28	
**Bank height**					
Behind dam	8	11.7	11.4	1.5	0.83
Not behind dam	79	12.1	11.9	0.52	
**BEHI**					
Behind dam	8	23.2	24.1	2.5	0.65
Not behind dam	79	24.4	27.2	0.69	

*Mann Whitney Rank Sum Test was used for Bank retreat and Bank height analyses. ANOVA was used for BEHI analysis.

### Riparian Land Cover and Soil Types

Most of the riparian areas adjacent to the study sites were forested with mature trees, primarily hardwoods, at the time of this study (Table 7). There was no relationship between BEHI values or bank heights with land cover in 2005/2007, as most of the riparian lands were forested. However, much of the land had been in agriculture for the past two centuries, so current geomorphic patterns may be a legacy from past land use.

**Table 7:** Land cover within the 153 m zone adjacent to BEHI study sites.

Land Use	Percentage
Forest	78.4
Open	15.3
Shrubland	5.5
Impervious	0.8

Agriculture was a major land use for the first half of the past century in the 3 study area counties within the watershed (Table 8). Both farm number and percentage of agricultural land decreased, with the greatest loss in the last half of the twentieth century. Counties in the Haw River watershed had 80–85% of the land area in agriculture at the beginning of the twentieth century. The 2007 agricultural census indicates this area had shrunk to 24–32% of each county [47]–[49].

**Table 8:** History of farming within the studied counties of the Haw River watershed.

Year		Alamance	Chatham	Orange
1910	Number of farms	2508	3640	1957
	% of county in agriculture	80	85	84
1950	Number of farms	2940	2977	2038
	% of county in agriculture	79	66	70
2007	Number of farms	753	1089	604
	% of county in agriculture	32	24	24

There were twenty one different soil types at the 87 BEHI study sites (Table 9). The most common soil type was Riverview Silt Loam, followed by Buncomb loamy fine sand.

**Table 9:** Soil types at BEHI study sites.

Soil ID	Soil Type	% slope	# sites
AdE	Appling, sandy loam, steep phase	20	2
Ba	Buncomb loamy fine sand, frequently flooded	1	14
BaE	Badin Nanford Complex	23	6
CbE	Cecil fine sandy loam, moderately steep phase	17	2
Cg	Congaree fine sandy loam, frequently flooded	1	2
ChA	Chewacla and Wehadkee soils, frequently flooded	1	1
Cp	Congaree fine sandy loam, frequently flooded	1	4
GaE	Georgeville silt loam, moderately steep phase	17	1
GbE3	Georgeville silt loam, severely eroded, moderately steep phase	20	1
GcE	Goldston slaty silt loam, moderately steep phase	17	5
GkE	Georgeville Badin complex	23	2
LbE	Lloyd loam, moderately steep phase	17	1
Mc	Mixed alluvial land, poorly drained	1	6
Md	Mixed alluvial land, well drained	1	6
NaD	Nanford Badin complex	5	1
RvA	Riverview silt loam, frequently flooded	1	28
TaD	Tirza silt loam, strongly sloping phase	30	1
WcE	Wilkes stony soils, moderately steep phase	17	4

## Discussion

The banks of the Haw River, located in the central Piedmont of North Carolina, are deeply incised (11.8 m mean bank height), with steep banks in the straight reaches, inside bends, and outside bends of the river channel. There is overhanging vegetation at the top of banks, with mass wasting, bank failures, and collapse of trees into the river. The high banks, BEHI indices and bank retreat indicate active erosion is occurring and the potential for future erosion is high. Over half of the study sites had a BEHI of moderate to high erosion potential, supporting observations that the banks along the Haw River in the study area are unstable. The average difference between bank height and bankfull height was 6.6 meters.

The slopes of the riparian areas were significantly correlated with the BEHI, bank height and bank retreat, primarily through an increase in bank height, BEHI and bank retreat as the slope of the riparian areas decreased (Tables 3 and 4). This suggests that river banks adjacent to riparian areas with low slope are more eroded and erodible than river reaches with more steeply sloped riparian areas.

The riparian areas with low slope are mostly alluvial (Table 9), likely including migrating soils from the surrounding agricultural uplands and deposited sediment from past floods. Gross floodplain sediment trapping potential has been shown to be a function of floodplain area, with larger floodplains having greater trapping potential [50]. If sediment migrating from the uplands was deposited in riparian areas with low slope, the deposited sediment would likely be more erodible than the original base. Riparian areas with high slope adjacent to the river would be less likely to have extensive sediment deposition as the waters carrying the sediment have comparatively more energy than areas which were more flattened.

Riparian sediment deposition from agriculture on the Haw is consistent with descriptions of sediment deposition layers between 1 and 6 m in depth which have been found adjacent to other streams of the eastern United States. A history of row crop agriculture has caused a loss of 14 cm of topsoil from the North Carolina Piedmont, with sediments migrating from the upland fields to riparian areas and associated rivers and streams [3], [4]. These sediment deposit layers are likely an outcome of land clearing for development, agriculture within the watershed, deposition behind dams, and deposits from past flood events, showing the potential for substantial sediment migration to storage areas along riparian river borders [17]. Recent and continued sediment deposition from current agriculture into riparian areas has been documented in the upper Midwest of the United States [51], similarly to patterns on the Haw River.

The riparian areas were mostly forested at the time of the study, with root density and root depth ratios negatively correlated with bank height, but not with bank retreat. Although mature trees were present in the riparian zone throughout the length of the study site, roots rarely extended to the base of the bank. Root density in river banks has been shown to reduce scour through both mechanical root reinforcement and matric suction [52]. However, on the Haw, the bank height frequently exceeded rooting depth (Table 2). The absence of roots at the base of the river bank would mean that roots are not present to provide reinforcement and protection from shear. In contrast to the protective effect of roots on bank height, the lack of correlation with bank retreat suggests vegetation provided no protection from erosion of the bank toe slope at the river's edge, a significant concern in river management [53].

The influence of dams on river geomorphology has been well documented [10], [12], [13]. In the present study, dams were lower than the heights of banks measured in the study area and the eroded bank areas extended well upriver beyond dam impoundments. The highest dam currently on the Haw is the Saxapahaw Dam, with a height of 9.1 m. The mean height for all dams in the studied segment was 2.9 m, including both historical and present dams. Yet the mean bankfull measurement was 5 m, and the mean bank height was 11.8 m, higher than all the dams. If bank erosion was primarily through legacy sediments from dam impoundments, it would be expected that the bankfull and bank heights would not exceed the heights of the dams. The Haw River bed is rocky, lined with rocks from 0.2 m diameter to large boulders and bedrock, so the contribution to bank heights from erosion of the river bed is likely to be minimal. Comparison of the bank heights, BEHI values and bank retreat upstream of the legacy dam sites with other study sites showed no difference between locations (Table 6). Although low dams can have significant ecological effects in many cases, the impact to higher order rivers such as the Haw appears to be small.

The lack of a correlation between bank height and the BEHI with bank retreat suggests that different but related processes are occurring. Bank retreat often occurs following bank failure and collapse, with the river bank collapsing downward towards the water's edge then eroding away as the river flows past. River bank stability can be highly variable, with many factors contributing to the structural integrity. The negative correlation between bank height and BEHI with river width suggests that many of the tall banks are stable, reflective of the wide range of conditions present in the river corridor. However, both high banks and bank retreat were correlated with low riparian slope, suggesting this condition increases risk from both patterns of erosion [54].

Geomorphic patterns of the Haw River are consistent with conceptual models describing changes in river geomorphology following disturbance such as the removal of a small dam. Following dam removal, the sequence would be: a) lowered water surface, b) degradation, c) degradation and widening d) aggradation and widening, ending with e) quasi-equilibrium [32], [55], [56]. This process usually happens quickly after removal of small dams, reaching quasi-equilibrium within a few years. A similar sequence of geomorphological changes following disturbance seems to be occurring on the Haw River, with the river currently at stages b, c and d.

Disturbances on the Haw River include historical dams which are no longer in place, three dams which are currently in place, a history of row crop agriculture throughout the watershed and upstream urbanization. This is similar to the history of dams and land use on other rivers of the Piedmont and around the world [18], [57]. Erosion of legacy sediments from agriculture followed by urbanization is has also been documented on a smaller stream in the Piedmont region, leaving similarly incised banks [36].

Reaching dynamic equilibrium does not always occur quickly. Following an eruption of Mount St. Helens in 1857, stable floodplains and re-vegetation of riparian zones had not yet been re-established at the time of the 1980 eruption [33]. In this case, dynamic equilibrium had not been reached over a century after the earlier disturbance. Similar patterns have been observed in other regions [5], [58], [59]. For the Haw River, re-connection with flood plains and equilibrium will likely take centuries or longer, if it all, compared with the decadal time scale described with dam removal.

**Figure 3:**
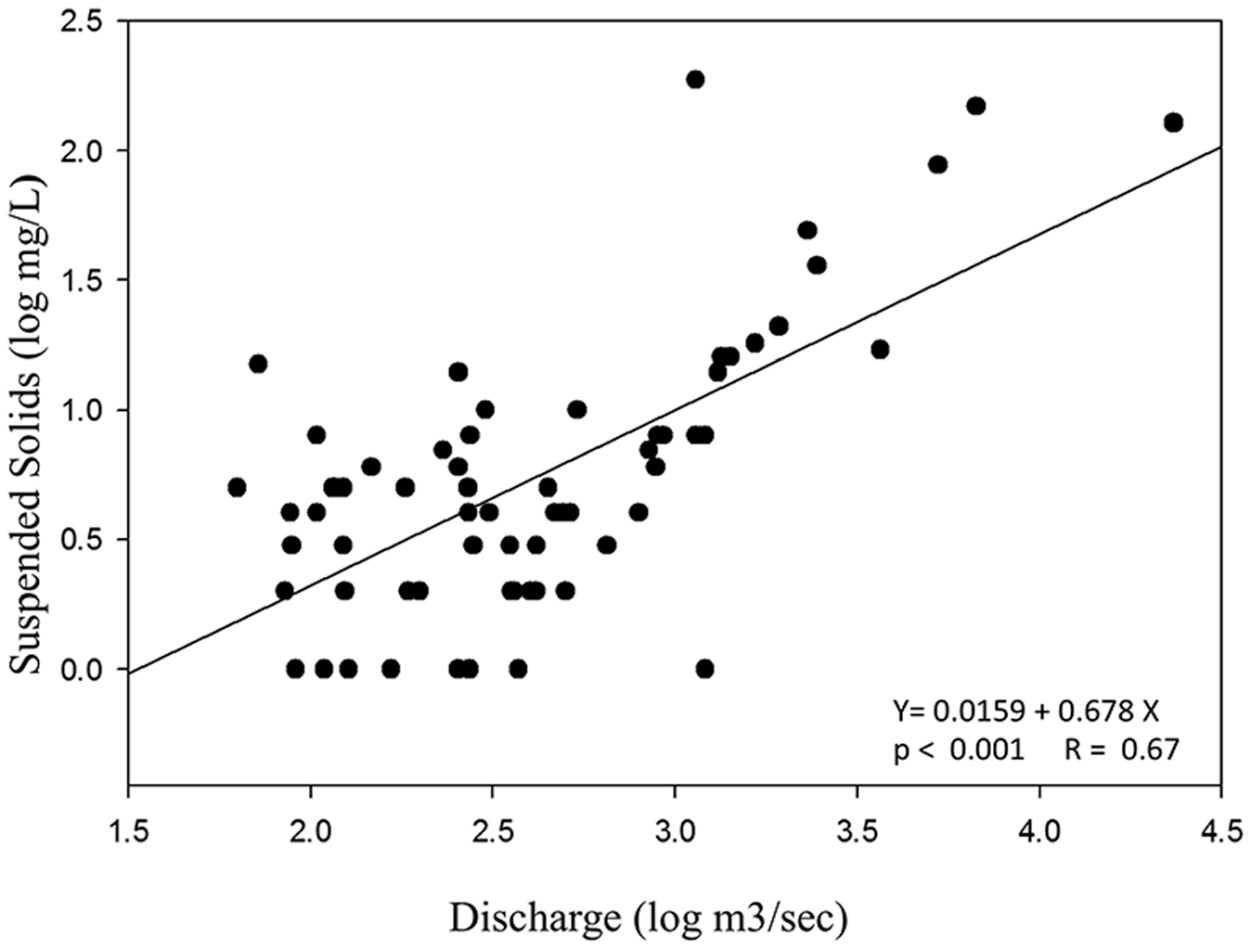
Relationship between log of river discharge and log of total suspended solids in the Haw River.

In addition to the effects from dams and past agriculture, changes in flow can affect soil loss from erosion and sediment load. Data from a U.S.G.S. monitoring station just downstream of the study area were examined from 2005–2010, with flow and sediment measures collected on the same day [37], [60]. As flow increased, so did sediment loading to the river, as reflected in the concentration of suspended solids (Figure 3). River discharge ranged from 2 m^3^/sec to 659 m^3^/sec, averaging 31 m^3^/sec. The suspended solids in the river ranged from 1 mg/L to 187 mg/L, averaging 15 mg/L. As the energy in the river increased with flow, the concentration of suspended solids also increased exponentially, a relationship well documented in the literature [2]. As the region upstream of the study area continues to urbanize with increasing impervious surface, high flow events are likely to also increase with increasing erosion, a significant management concern.

Sediment loading from bank erosion to the Haw River will also contribute P. The analyses of riparian soil indicated approximately 3759 kg (8286 lb.) of P enters the river annually from sediment. This is about 1.5% of the total target P load from non-point sources in the Haw River flowing to Jordan Reservoir (of a targeted 106,884 kg), and potentially 6% of the total P load to the Haw River from river bank erosion (the study area was about 25% of the river length). Currently this is an unaccounted non-point source of P, with no Best Management Practices or nutrient loading targets assigned to this P source [34].

Like many other high order rivers globally, the geomorphology of the Haw River in North Carolina is changing following agricultural and other disturbances, with significant sediment and P loading to the river. The river is undergoing a process of reshaping with river widening and the formation of very high river banks. Occasional high flow events further contribute to erosion with the concentration of suspended solids in the water column increasing with discharge (Figure 3).

Future study of the Haw River and similar systems could be directed at understanding the patterns of erosion seen on this river. One major area of investigation would be to determine the origin and age of sediments deposited along the river, especially those in low slope areas which are experiencing the most erosion. Another study would be to evaluate the impacts of past land use and land cover on contemporary geomorphology, flow and erosion. Comparison with commonly used models for erosion estimates would also be valuable.

## Conclusion

Few studies on the patterns of bank erosion and hydrogeomorphic change following disturbance have been published for high order rivers such as the Haw River. However, differences in geomorphology have been described between high and low order segments of rivers, with factors impacting the river attributes changing throughout the river's length. These studies show processes and attributes of low order rivers may not be applicable to larger river systems [52].

For most studies estimating erosion and erosion potential of larger rivers, a modeling approach has been used. Common methods are the Universal Soil Loss Equation and the Soil and Water Assessment Tool [61], [62]. In contrast, field measurements of large systems are seldom the major approach used to identify factors influencing erosion and geomorphic change.

For the Haw River, regions of high erosion potential (as indicated by the BEHI and bank height) are negatively correlated with river width, suggesting regions high BEHI values and bank heights are have narrow river width and are relatively stable. On this river, erosion as measured by bank retreat is independent of most physical features such as bank height, BEHI, bank angle and bankfull height, suggesting different erosional processes are occurring. Although these physical features are often used to predict erosion potential, such as through use of the BEHI, that does not appear to be the case in this study. Independence of bank physical features with bank retreat has been observed in a few other systems [65].

Areas with low riparian slope (<2%) appear to have the highest erosion risk, experiencing high bank heights, BEHI values and bank retreat. This relationship is in contrast to some common models used for erosion estimation, which rank high slope as an erosion risk factor [61], [62]. In the Haw River system, a history of extensive soil loss from upland agriculture suggests erosion of agricultural legacy sediments deposited on areas with low slope beside the river has occurred, with high erodibility. Increasing sediment concentration with flow suggests the river is still changing, reshaping as an outcome of past and present disturbance. The potential for further impairment as upstream urban development increases should be a management concern for the river health and water quality.

Rate of bank retreat and river widening is similar to the rate reported for other high order rivers. In rivers of southern Minnesota, in the Midwest of the United States, LIDAR studies have indicated widening rate of 0.57–5 m/yr since European settlement [63]. In our study, the Haw has widened 0.38 m/yr, slightly less than in Minnesota. But both studies indicate the rivers are not at equilibrium.

The Haw River is an unstable system, with river bank erosion and geomodification potential influenced by disturbance, riparian slope and episodes of high flow. The greatest erosion, measured by bank height and bank retreat occurred in regions with low riparian slope, usually with alluvial soils, suggesting erosion of deposited sediments. Historical dams were not a significant factor in influencing current conditions on the Haw River. This study provides a model for high order rivers, identifying factors driving erosion and changes to channel morphology which will help in management of these and other high order river systems.
